# Pulmonary Rehabilitation Nursing Interventions Promoting Self-Care in Elderly People with Chronic Obstructive Pulmonary Disease (At Home)

**DOI:** 10.3390/healthcare13172176

**Published:** 2025-08-31

**Authors:** Susana Salvado, Eugénia Grilo, Helga Henriques, Isabel Ferraz, Filomena Gaspar, Cristina Baixinho

**Affiliations:** 1 Nursing Research, Innovation and Development Centre of Lisbon, 1900-160 Lisboa, Portugal; hrafael@esel.pt (H.H.); ipereira@esel.pt (I.F.); mfgaspar@esel.pt (F.G.); crbaixinho@esel.pt (C.B.); 2 Unidade Local de Saúde da Cova da Beira, 6230-411 Fundão, Portugal; 3 Interdisciplinary Research Unit on Building Functional Ageing Communities, Escola Superior de Saúde Dr. Lopes Dias, Instituto Politécnico de Castelo Branco, 6000-767 Castelo Branco, Portugal; eugenia@ipcb.pt; 4 Escola Superior de Enfermagem de Lisboa, 1900-160 Lisboa, Portugal; 5 Center of Innovative Care and Health Technology (ciTechCare), 2414-016 Leiria, Portugal

**Keywords:** elderly person, self-care, chronic obstructive pulmonary disease, respiratory rehabilitation, nursing, home

## Abstract

**Background/Objectives**: Pulmonary rehabilitation is recognised as one of the most cost-effective interventions. However, patients’ adherence to these programmes remains a challenge. This systematic literature review aimed to describe pulmonary rehabilitation interventions carried out by nurses that promote self-care in elderly people with COPD (at home). **Methods**: The exploratory literature search was conducted to support the development of the research question and the PICO strategy. The criteria for eligibility were determined for participants, interventions, comparators, and outcomes. Research was conducted in the CINAHL, SCOPUS, and MEDLINE databases and that covered publications up to 31 December 2024 with no temporal limit identified, eight articles that included cohort studies, randomised controlled trials (RCTs), and quasi-experimental studies that met the quality standards established by JBI. **Results**: The identified interventions include health education, breathing training and physical exercise, the development of tailor-made plans with monitoring and follow-up, psychological support, and oxygen therapy management. **Conclusions**: The results highlight the importance of tailor-made interventions that can enhance self-care in elderly people with COPD in a home setting, as well as key components of respiratory rehabilitation.

## 1. Introduction

Population ageing is accelerating worldwide and has become a significant trend in Europe over recent decades [1,2]. In the European Union, the proportion of individuals aged 65 or over has increased from 16% in 2003 to 21% in 2023 [3]. In Portugal, this demographic trend is equally evident: in 2022, the ageing index—comparing the population aged 65 or over with those aged 0 to 14—reached 185.6 elderly individuals per 100 young people, up from 181.3 in 2021 [4]. The increase in the elderly population applies considerable pressure on healthcare and social security systems [5].

Chronic obstructive pulmonary disease (COPD) is a widespread condition among older people [6,7] and is one of the leading causes of morbidity and mortality worldwide [8,9,10]. It is defined as a prevalent, preventable, and treatable disease characterised by persistent respiratory symptoms and airflow obstruction caused by alveolar and/or airway alterations resulting from prolonged exposure to harmful particles or gases, as well as genetic predispositions [8].

People that suffer from COPD often face physical, psychosocial, and emotional hardships that limit their ability to manage self-care, including trouble breathing, eating, dressing, and maintaining personal hygiene [11]. Self-care is a central concept in Orem’s theory [12] and refers to an individual’s ability to autonomously manage their own needs [13]. In Orem’s self-care theory, self-care is understood as a learned and intentional behaviour aimed at maintaining life, health, and well-being [12]. A self-care deficit takes place when one’s self-care capacity is insufficient to meet their necessary requirements, making nursing care essential [12,13]. All nursing interventions aim to promote one’s active participation in their own self-care, ensuring the continuous fulfilment of their self-care needs [12] and emphasising the importance of maintaining independence in daily self-care activities [14].

Pulmonary rehabilitation (PR) may be one of the most cost-effective treatment methods [8,15,16] and is a key pillar of non-pharmacological interventions for people with COPD [10,17]. It stands out as a therapy that not only reduces symptoms [18] but also offers a favourable cost–benefit ratio [15]. PR is described as an essential care element in individuals with chronic respiratory disease [18]. Participating in PR reduces dyspnoea, increases exercise capacity, improves health-related and emotional-related quality of life, and provides social support [18]. PR also contributes to reducing hospital admissions and the risk of mortality after hospitalisation in people with COPD [18].

Although not limited to the mentioned therapies, the therapies included in PR involve physical training, education, and behavioural change strategies, all designed to improve the physical and psychological well-being of individuals with chronic respiratory diseases and promote adherence to practices that support their health in the long term [19].

The literature documents assigned obstacles to the acceptance and participation of individuals in PR interventions [8], including travel requirements [18,20], transportation challenges [8] and the limited availability of services in contexts where dyspnoea and reduced mobility are common [20]. Consequently, home-based PR interventions can improve adherence amongst people with COPD [21] and provide a practical alternative that is comparable to conventional outpatient rehabilitation programmes [8,22].

A recent systematic review and meta-analysis of randomised controlled trials on the effectiveness of pulmonary rehabilitation in older adults with COPD [23] concluded that more comprehensive pulmonary rehabilitation programmes should be made available for this population, whether in community hospitals, large public hospitals, or at home. The importance of evidence-based nursing practices in guiding interventions and treatments for older adults with respiratory disorders was also emphasised in another recent systematic review [24]. Furthermore, according to a recent study [7], the long-term survival effects of continuous nursing interventions, combined with guided respiratory training in elderly patients with COPD, still warrant further investigation.

Given these challenges, it is essential to optimise the outcomes of healthcare interventions for patients and to reduce the overall burden on healthcare systems worldwide [25]. Therefore, this systematic review aimed to describe pulmonary rehabilitation interventions carried out by nurses that promote self-care in elderly people with COPD at home.

## 2. Materials and Methods

### 2.1. Study Design

The systematic literature review was chosen, keeping in mind the study’s objective and the state-of-the-art phenomenon under analysis. This systematic review followed the recommendations outlined by the JBI criteria, ensuring research rigour in study design, bibliographic research, and data analysis [26]. The study protocol was previously registered and published in the PROSPERO database (CRD42022308062).

### 2.2. Research Question and Eligibility Criteria

The research question that was formulated using the PICO search strategy was “What are the pulmonary rehabilitation interventions carried out by nurses that promote self-care in elderly people with COPD at home?”

Based on the research question, eligibility criteria were defined and used in the process of searching, selecting, and analysing the scientific evidence (Table 1).

The selected articles were limited to those for which full-text access was available, regardless of the publication’s access policy, and were published up until 31 December 2024 in Portuguese, English, or Spanish.

This review opted to exclude studies that used devices or technological applications in respiratory rehabilitation. The decision was made to ensure that the analysed interventions were exclusively those carried out in a home setting with face-to-face interaction. Albeit technology plays an increasing role in respiratory rehabilitation, priority was given to the analysis of strategies that could be implemented in an approachable manner across different contexts without the need for advanced technological resources.

### 2.3. Research Strategy

The research was conducted in the following databases: MEDLINE (via PubMed) (Table 2), CINAHL (Table 3), and SCOPUS.

The preliminary research strategy combined natural language terms with indexed language, and it is important to note that the indexed terms were appropriately adapted to the specifics of each database, particularly standardised medical descriptors (MeSH) or standardised subject headings (subject headings). To structure the research, the terms were combined with Boolean operators OR and AND, following the PICO approach.

### 2.4. Data Extraction, Quality Appraisal, and Data Synthesis

Data extraction and coding were carried out independently by two reviewers. Any discrepancies were addressed by consulting with a third reviewer.

After identifying relevant articles in the selected databases, they were exported in “.ris” format to the Rayyan web application (available online https://www.rayyan.ai) to facilitate collaboration amongst reviewers during the study selection process. Initially, duplicates were removed. After that, the titles and abstracts were examined, and relevant studies were selected for full-text review.

After an initial review of titles and abstracts, studies that were deemed to be irrelevant were excluded. Full texts were read and examined according to the inclusion and exclusion criteria for potentially eligible studies.

For the selected studies, two researchers extracted key study characteristics into a table independently, including the study’s identification (author, year of publication, and country of origin), study design, objective, participants, and interventions. Data extraction was conducted by two researchers. Any discrepancies were addressed by consulting with a third reviewer.

Both the methodological quality and risk of bias of the included studies were independently assessed by two reviewers using JBI’s critical appraisal tools. Any discrepancies were addressed by consulting with a third reviewer.

## 3. Results

**Figure 1:**
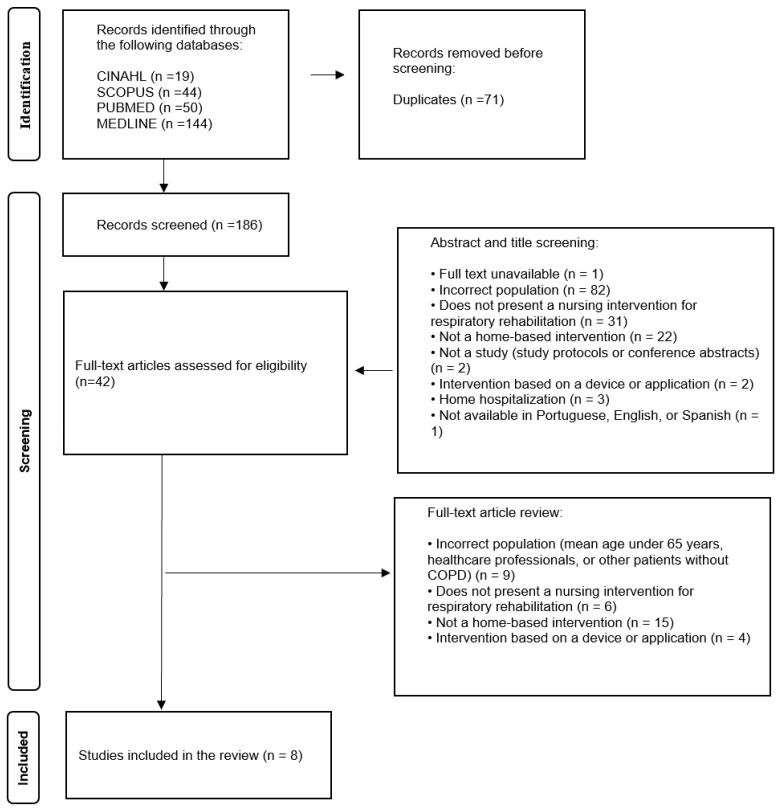
Flowchart of article selection.

The database searches resulted in 257 articles (Figure 1). After removing duplicates, 186 articles remained. Title and abstract screening led to the exclusion of 144 articles. A total of 42 articles were read in full, of which 34 were excluded (Figure 1). As a result, eight articles were included in this study (Table 4).

The methodological quality of the included studies was assessed using JBI’s critical appraisal tools. The assessment included cohort studies (Table 5), randomised controlled trials (RCTs) (Table 6), and quasi-experimental studies (Table 7).

The studies were analysed independently by two reviewers, and any discrepancies were addressed by consulting with a third reviewer.

The analysed cohort studies showed variations in methodological quality. Study [30] met all established methodological criteria, ensuring good reliability of its results. However, study [29] showed weaknesses in essential criteria (items 4, 5, and 10), suggesting the need for cautious interpretation of its results.

The clinical trials included in the review, namely [27,28], revealed methodological weaknesses, especially in the blinding of subjects and evaluators (items two, four, and five). This limitation may compromise the internal validity of the studies, requiring caution in extrapolating the results.

The quasi-experimental studies showed variable methodological quality. Studies [32,33] met almost all methodological criteria, suggesting good bias control. By contrast, studies [31,34] showed weaknesses in bias control (item eight) and methodological clarity (item two).

Despite some limitations, most quasi-experimental studies can be considered methodologically robust.

This systematic review identified and analysed eight studies investigating interventions in pulmonary rehabilitation for older adults with COPD in a home setting. The identified interventions were categorised into five main areas (Table 8). When it was possible to clearly attribute an intervention to nursing professionals, a check mark (✓) was placed in the corresponding cell. When the intervention was delivered within a multidisciplinary team and the role of the nurse was not specifically distinguishable, the cell was marked with an “M”, indicating a multidisciplinary delivery involving nurses. This approach ensures clarity while respecting the methodological limitations of the primary studies.

## 4. Discussion

As identified in the analysis of the included studies, in studies [29,30], the interventions were carried out within the scope of multidisciplinary pulmonary rehabilitation programmes. However, it was not possible to clearly determine which interventions were specifically delivered by nurses. The lack of this distinction in the articles limits the direct attribution of certain practices to the nursing profession. Nevertheless, all the interventions analysed in this review were described as being performed by nurses in at least one of the included studies. Therefore, the following section presents a discussion of the identified interventions based on the available evidence.

To define pulmonary rehabilitation programmes for older adults with COPD, it is first necessary to identify the relevant rehabilitation interventions. According to Orem’s nursing theory, nursing interventions aimed at individuals with COPD should promote the development of self-care skills, specifically the following [16]: cognitive (knowing), instrumental (knowing how to do), and personal (knowing how to be). The findings of this systematic review identify five key areas in the development of programmes for older adults with COPD: health education, breathing training and physical exercise, personalised planning with monitoring and follow-up, psychological support, and oxygen therapy management. These findings align with the existing literature, which highlights the effectiveness of these approaches in promoting self-care and improving quality of life [8,35,36,37].

Health education is widely recognised as a fundamental pillar in the treatment of COPD, giving patients the necessary knowledge and skills for effective self-management of the disease [17]. The review identified five key topics commonly addressed in COPD education: understanding the condition, correct use of medication, smoking cessation, symptom and exacerbation management, and nutrition.

The incorrect use of inhalers is common, and it compromises therapy effectiveness, making continuous education by specialised nurses essential [36]. Recent publications highlight that education on the correct use of pharmacological therapy, particularly inhalers, is a priority intervention, as incorrect use is widespread and negatively impacts treatment efficacy [36,37]. This education should be ongoing, ideally provided during each visit, and it should include demonstrations and technique verification [8]. Recent studies suggest that this intervention should be carried out by specialised nurses, which would significantly improve patients’ performance [36].

The reviewed studies (approximately 87.5%) reinforce the need for interventions focused on the early identification of exacerbations and symptom management. Recent publications highlight these aspects as fundamental for reducing exacerbations and preventing hospitalisation, underlining their importance in minimising disease progression and healthcare burden [38].

Smoking cessation was also widely addressed in the reviewed studies (approximately 87.5%), as around 40% of people with COPD continue to smoke even after being diagnosed [39]. Nurses play a crucial role in supporting smoking cessation, using strategies and setting goals with patients [40].

Malnutrition is common among people with COPD [41,42] and it is associated with a worse prognosis [8]. A significant proportion of the reviewed studies (50%) highlighted the importance of nutritional counselling. According to recent studies, this should focus on increasing caloric and protein intake to improve nutritional status and quality of life in people with COPD [41].

Not only that, but still within the scope of health education, some studies (25%) included the importance of educating family members and caregivers.

The decline of physical activity and reduced exercise tolerance pose a challenge for individuals with COPD [43], as inactivity leads to a vicious cycle of exercise deconditioning, worsening dyspnoea, and muscle weakness [21,44].

Respiratory training and physical exercise are widely acknowledged in the literature as essential components of pulmonary rehabilitation [8] and were identified in the majority of the analysed studies. This review identified the following effective interventions: endurance (aerobic) and strength (anaerobic) training, functional respiratory retraining techniques, and energy conservation techniques.

The recent literature points to aerobic and strength training as essential strategies in pulmonary rehabilitation [35], as did some other studies (37,5%). Guided exercise sessions are recommended at least twice a week and may include a variety of programmes, e.g., resistance training, strength training, and interval training. Upper and lower limb exercises should be included, as well as walking exercises; flexibility exercises, inspiratory muscle training, and neuromuscular electrical stimulation may also be incorporated [8].

The most recent Global Initiative for Chronic Obstructive Lung Disease reinforces the importance of structured, supervised, and individualised pulmonary rehabilitation programmes [45]. These should ideally include exercise sessions at least twice per week, combining endurance and strength training, inspiratory muscle training, flexibility exercises, and, when appropriate, neuromuscular electrical stimulation. GOLD 2025 also emphasises the need to tailor these programmes to patients’ physical capabilities, preferences, and social contexts [45]. Furthermore, it recognises the role of home-based rehabilitation as a practical and equally effective alternative to traditional outpatient settings, particularly in populations with reduced mobility or limited access to healthcare services [45].

Functional respiratory retraining techniques such as diaphragmatic breathing and pursed-lip breathing are essential for optimising adaptation to exercise [17], with 50% of the studies highlighting their importance. On the other hand, as exacerbations in individuals with COPD often involve mucus hypersecretion, managing sputum retention is crucial [46]. This issue was also addressed in some studies (n = 4; 50%) that highlighted the importance of airway clearance techniques. Most international guidelines recommend the use of Positive Expiratory Pressure (PEP), which has achieved the greatest consensus [45]. In clinical practice, the recent literature indicates that the most frequently used techniques include the Active Cycle of Breathing, Positive Expiratory Pressure (PEP), Oscillating Positive Expiratory Pressure, Forced Expiratory Technique, and Postural Drainage, Percussion, and Vibration [46].

Individuals with COPD often experience severe dyspnoea, which significantly impacts their ability to perform activities of daily living (ADLs) [47]. In this regard, approximately 50% of the articles emphasised energy conservation techniques in their studies. The recent literature suggests that training in energy conservation techniques is a valuable approach to improving both the tolerance and execution of these ADLs [47].

The results of this study also highlight the importance of a tailor-made plan or at least one tailored intervention, as emphasised in all the articles. The literature points to the need for tailor-made programmes [35,48] to maximise the benefits of pulmonary rehabilitation [8], tailored to the individual needs and preferences of patients [48].

The importance of monitoring was also highlighted in the reviewed articles (50%). In this context, the continuous monitoring of people with COPD should include the assessment of symptoms, exacerbations, objective measurements through spirometry, the 6 min walk test or resting oxygenation assessment [8], as well as continuous home follow-ups and phone contact [28,34].

Anxiety and depression are common in people with COPD, worsened by the fear of exacerbations [36]. In this study, 75% of the articles addressed the relevance of psychological support. The recent literature states that therapies such as cognitive–behavioural interventions and counselling have been shown to be effective in reducing these symptoms, promoting greater emotional stability [36].

Oxygen therapy is often necessary in the treatment of COPD [49], and its correct administration is crucial to avoid complications [50]. The management of oxygen therapy in the pulmonary rehabilitation programme was also addressed by 37.5% of the articles. The recent literature states that nurses play a key role in monitoring the safe use of oxygen, ensuring its effectiveness and intervening in cases of complications [50]. And while there are no studies demonstrating the significant effect of supplemental oxygen use during physical training, many physicians provide oxygen for safety reasons and to improve patient comfort, as it reduces dyspnoea during physical training [48].

The reviewed studies show that nursing interventions in home-based pulmonary rehabilitation include multiple strategies, health education being one of the most frequently mentioned components. Recent research recommends health education to be an essential part of rehabilitation programmes [17]. Moreover, the findings of this review reinforce that the combination of multiple nursing intervention strategies tailored to the individual needs of elderly people with COPD can enhance treatment adherence, improve functionality, and reduce disease-related complications, ultimately improving their self-care.

The studies included were conducted in various countries, namely Turkey, the United States, Ireland, Italy, Australia, and Hong Kong. Most studies do not provide detailed descriptions of the healthcare systems or access mechanisms, and this diversity should be considered when interpreting the results.

Even though this review identified pulmonary rehabilitation interventions for people with COPD, several limitations should be considered, namely the inclusion of articles that were only published in specific databases, which may have limited the scope of the review, potentially excluding relevant studies available in other sources.

Additionally, this review excluded studies involving devices or mobile applications in order to maintain a clear focus on face-to-face nursing interventions delivered in the home setting. While such technologies may provide relevant benefits—particularly in remote monitoring and telehealth—their inclusion could have introduced considerable methodological variability and compromised the homogeneity of the analysis, which was centred on direct, hands-on nursing care.

Another limitation is the methodological diversity of the included studies, which presented varied designs, samples of different sizes, and heterogeneous approaches in the reviewed interventions. This heterogeneity made it difficult to draw direct comparisons between the studies and the generalisation of results.

Despite these limitations, the results of this study provide a relevant contribution to the knowledge of nursing interventions in pulmonary rehabilitation at home as a central strategy to promote self-care in elderly people with COPD. The identification of specific practices such as health education, respiratory training, physical exercise, tailor-made interventions with monitoring and follow-up of the elderly person, psychological support, and the management of oxygen therapy offers a valuable guide for nurses working in community settings.

Although recent studies have focused on promoting self-care in people with COPD [51], the World Health Organization (WHO) [52] emphasises the need for further research in this area, and the recent literature [9] advocates for the implementation of self-care adherence programmes for elderly people with COPD. This review contributes to advancing the field by proposing nursing interventions for pulmonary rehabilitation for elderly people with COPD. This review may guide the creation of more accessible and effective pulmonary rehabilitation programmes.

## 5. Conclusions

This systematic review identified and described home-based nursing interventions aimed at individuals with COPD, with a potential impact on self-care support. The main strategies include health education, respiratory training, physical exercise, personalised plans with monitoring and follow-up, psychological support, and the management of oxygen therapy. These interventions have been widely mentioned in the literature as strategies that are used to promote self-care and improve disease management, although the reviewed studies show differences in the adopted methodologies and approaches.

For the clinical practice, this review provides a foundation for structuring home-based pulmonary rehabilitation programmes, highlighting essential components that should be considered in the care of elderly people with COPD. Empowering nurses to implement these strategies and adapting interventions to individual needs are key aspects for optimising the provided care.

Additionally, it was found that the follow-up periods were often short, preventing the long-term sustainability of the effects of the interventions from being assessed.

In terms of research, the results highlight the need for studies that rigorously evaluate the effectiveness of these interventions for elderly people with COPD through randomised controlled trials (RCTs) with representative samples and appropriate follow-up periods to establish more robust recommendations for clinical practice, thereby strengthening the body of evidence on the effectiveness of nursing interventions in self-care for elderly people with COPD.

Moreover, it is essential that future studies identify in detail the specific needs of elderly people with COPD living at home. This knowledge will enable the design of increasingly personalised and effective pulmonary rehabilitation programmes that can meet the demands of an ageing society.

## Figures and Tables

**Table 1:** Eligibility criteria.

Inclusion Criteria	Exclusion Criteria
Participants: Elderly people (with a mean age of 65 years or older in the articles) diagnosed with COPD.	Studies that include people with other respiratory diseases such as asthma or that simultaneously cover people with COPD and other conditions.
Intervention: Studies in which at least one pulmonary rehabilitation intervention is carried out by a nurse at home.	Studies in which the interventions involve the use of devices or applications. Studies referring to home hospitalisation settings.
Comparator: Not applicable, as the adopted methodology allows the integration of studies with different methodological designs, thus not ensuring comparison between them.	
Outcomes: Studies in which the rehabilitation intervention in elderly individuals promotes: - Self-care; - The performance of daily life activities; - Daily life, instrumental activities; - Improved functionality of the elderly person.	

**Table 2:** Research strategy used in the MEDLINE PubMed database.

	Search Strategy	N
S1	“Pulmonary Disease, Chronic Obstructive” OR “Pulmonary Emphysema” OR “Bronchitis, Chronic” OR “Lung Diseases, Obstructive” OR “Respiratory Tract Diseases” OR “copd” OR “chronic air flow obstruction” OR “Chronic Obstructive Airways Disease”	134,196
S2	“Aged” OR “Geriatrics” OR “Gerontologic Nursing” OR “Gerontologic Care” OR “Older Adult Care” OR “Elderly” OR “Senior” OR “Elder” OR “Older”	6,716,808
S3	“Ambulatory Care” OR “Ambulatory Care Information Systems” OR “Ambulatory Care Nursing” OR “American Academy of Ambulatory Care Nursing” OR “Community Programs” OR “Community Health Services” OR “Community Health Nursing” OR “Home Health Care” OR “Home Health Care Information Systems” OR “Home Respiratory Care” OR “Primary Health Care” OR “Primary Nursing” OR “Home Care Services” OR “Home Health Nursing” OR “Nurses, Community Health” OR “Primary Care Nursing”	302,198
S4	“nurs*”	1,232,767
S5	“Abdominal Exercises” OR “Aerobic Exercises” OR “American Association for Respiratory Care” OR “Association of Rehabilitation Nurses” OR “Breathing Exercises” OR “Drainage, Postural” OR “Endurance Training” OR “Exercise Tolerance” OR “Exercise” OR “Exercise Intensity” OR “Exercise Positions” OR “Exercise Therapy” OR “High-Intensity Interval Training” OR “Physical Education and Training” OR “Rehabilitation” OR “Warm-Up Exercise” OR “Rehabilitation Exercise” OR “Rehabilitation Nursing” OR “Rehabilitation Research” OR “Rehabilitation Patients” OR “Rehabilitation, Pulmonary” OR “Research, Rehabilitation” OR “Resistance Training” OR “Respiratory Therapy” OR “Telerehabilitation” OR “Therapeutic Exercise” OR “respiratory exercises” OR “Exercise training” OR “Exercise Programs” OR “Pulmonary rehab*” OR “Education” OR “Education, Pharmacy” OR “Health Education” OR “Education, Nursing” OR “Patient Education” OR “Education, Respiratory Therapy” OR “Patient Education as Topic” OR “Support, Psychosocial” OR “Psychosocial Aspects of Illness” OR “Rehabilitation, Psychosocial” OR “Psychosocial Care (Saba CCC)” OR “Psychosocial Support Systems” OR “Psychiatric Rehabilitation” OR “Psychosocial Intervention” OR “Nutritional Support” OR “Nutrition Therapy (Iowa NIC)” OR “Diet Therapy” OR “Diet” OR “Home Nutritional Support” OR “Nutritional Assessment” OR “Nutrition Support (Iowa NIC)” OR “Nutrition Therapy”	3,454,187
S6	“Self-Care” OR “selfcare” OR “selfcare” OR “Self-Care Deficit (Saba CCC)” OR “Self-Care Component (Saba CCC)” OR “Self-Care Assistance: Dressing-Grooming (Iowa NIC)” OR “Self-Care Assistance: Bathing-Hygiene (Iowa NIC)” OR “Self-Care Agency OR Self-Care: Instrumental” OR “Activities of Daily Living (Iowa NOC)” OR “Self-Care: Toileting (Iowa NOC)” OR “Self-Care: Parenteral Medication (Iowa NOC)” OR “Self-Care: Oral Hygiene (Iowa NOC)” OR “Self-Care: Activities of Daily Living (Iowa NOC)” OR “Activities of Daily Living” OR “Instrumental Activities of Daily Living Alteration (Saba CCC)” OR “Instrumental Activities of Daily Living (Saba CCC)” OR “Activities of Daily Living Alteration (Saba CCC)” OR “Activities of Daily Living (Saba CCC)” OR “Functional Status” OR “Geriatric Functional Assessment” OR “Geriatric Assessment” OR “functional*” OR “Functional capacity” OR “Physical Functional Performance”	2,325,775
	S1 AND S2 AND S3 AND S4 AND S5 AND S6	47

**Table 3:** Search strategy used in the CINAHL Complete database.

	Search Strategy	N
S1	Pulmonary Disease, Chronic Obstructive OR Pulmonary Emphysema OR Bronchitis, Chronic OR Lung Diseases, Obstructive OR Respiratory Tract Diseases OR copd OR chronic air flow obstruction OR Chronic Obstructive Airways Disease	48,900
S2	Aged OR Geriatrics OR Gerontologic Nursing OR Gerontologic Care OR Older Adult Care OR Elderly OR Senior OR Elder OR Older	1,302,092
S3	Ambulatory Care OR Ambulatory Care Information Systems OR Ambulatory Care Nursing OR American Academy of Ambulatory Care Nursing OR Community Programs OR Community Health Services OR Community Health Nursing OR Home Health Care OR Home Health Care Information Systems OR Home Respiratory Care OR Primary Health Care OR Primary Nursing OR Home Care Services OR Home Health Nursing OR Nurses, Community Health OR Primary Care Nursing	232,598
S4	nurs*	1,017,896
S5	Abdominal Exercises OR Aerobic Exercises OR American Association for Respiratory Care OR Association of Rehabilitation Nurses OR Breathing Exercises OR Drainage, Postural OR Endurance Training OR Exercise Tolerance OR Exercise OR Exercise Intensity OR Exercise Positions OR Exercise Therapy OR High-Intensity Interval Training OR Physical Education and Training OR Rehabilitation OR Warm-Up Exercise OR Rehabilitation Exercise OR Rehabilitation Nursing OR Rehabilitation Research OR Rehabilitation Patients OR Rehabilitation, Pulmonary OR Research, Rehabilitation OR Resistance Training OR Respiratory Therapy OR Telerehabilitation OR Therapeutic Exercise OR respiratory exercises OR Exercise training OR Exercise Programs OR Pulmonary rehab* OR Education OR Education, Pharmacy OR Health Education OR Education, Nursing OR Patient Education OR Education, Respiratory Therapy OR Patient Education as Topic OR Support, Psychosocial OR Psychosocial Aspects of Illness OR Rehabilitation, Psychosocial OR Psychosocial Care (Saba CCC) OR Psychosocial Support Systems OR Psychiatric Rehabilitation OR Psychosocial Intervention OR Nutritional Support OR Nutrition Therapy (Iowa NIC) OR Diet Therapy OR Diet OR Home Nutritional Support OR Nutritional Assessment OR Nutrition Support (Iowa NIC) OR Nutrition Therapy	1,427,749
S6	Self-Care OR selfcare OR self-care OR Self-Care Deficit (Saba CCC) OR Self-Care Component (Saba CCC) OR Self-Care Assistance: Dressing-Grooming (Iowa NIC) OR Self-Care Assistance: Bathing-Hygiene (Iowa NIC) OR Self Care Agency OR Self-Care: Instrumental OR Activities of Daily Living (Iowa NOC) OR Self-Care: Toileting (Iowa NOC) OR Self-Care: Parenteral Medication (Iowa NOC) OR Self-Care: Oral Hygiene (Iowa NOC) OR Self Care: Activities of Daily Living (Iowa NOC) OR Activities of Daily Living OR Instrumental Activities of Daily Living Alteration (Saba CCC) OR Instrumental Activities of Daily Living (Saba CCC) OR Activities of Daily Living Alteration (Saba CCC) OR Activities of Daily Living (Saba CCC) OR Functional Status OR Geriatric Functional Assessment OR Geriatric Assessment OR functional* OR Functional capacity OR Physical Functional Performance	355,165
	S1 AND S2 AND S3 AND S4 AND S5 AND S6	50

**Table 4:** Objectives, participants, pulmonary rehabilitation intervention, and professionals involved in the studies.

Author, Year, and Country	Aim Study Design	Participants	Pulmonary Rehabilitation Intervention	Professionals Involved
		N (Age ± SD) (Men/Women)		
[27] (2002) Australia	To evaluate the usefulness of limited community-based care for patients with chronic obstructive pulmonary disease (COPD) after discharge from the hospital. RCT	- Number: 177 patients (intervention group: 84; control group: 93). - Age: Mean age 67.1 ± 6.2 years (intervention) and 66.7 ± 5.3 years (control). - Gender Distribution: 41 men (48.8%) and 43 women (51.2%) in the intervention group; 43 men (46.2%) and 50 women (53.8%) in the control group.	Patient Education on COPD: Comprehensive education on the disease, focusing on its pathophysiology, progression, and management strategies to enhance self-management and adherence to care. Smoking Cessation Counselling: Guidance on the importance of quitting smoking, including verbal and written support, as smoking cessation is critical to reducing disease progression and exacerbations. Management of Activities of Daily Living (ADLs): Education on safe and efficient strategies for performing ADLs, aiming to reduce physical strain and improve functional independence. Energy Conservation Techniques: Training in methods to conserve energy during daily activities, such as pacing, task prioritisation, and the use of adaptive tools. Medication Education and Adherence: Detailed instruction on the correct use of prescribed medications, particularly inhalers, and the importance of adherence to pharmacological treatment to control symptoms and prevent exacerbations. Physical Exercise: Guidance on incorporating safe and tailored physical exercises to improve respiratory function and overall endurance, aligned with the patient’s limitations. Early Recognition of Complications: Education on identifying early signs of complications or exacerbations that require medical intervention, promoting timely care-seeking behaviour.	Community nurses
[28] (2005) United States	The study aimed to investigate the effectiveness of increasing access to selected components of pulmonary rehabilitation—namely, patient education, self-management skill improvement, and enhanced follow-up—through nurse-assisted home care. RCT	- Number: A total of 151 patients completed the study. - Age: Mean age was 69 ± 8.2 years. - Gender: 56.9% were women.	Reassessment of Symptoms and Pharmacological Therapy: Conducted at the time of discharge to ensure the patient’s treatment plan is optimised and aligned with their clinical status. Patient Education: Focused on the disease (COPD), including symptom recognition and the appropriate use of prescribed medications. Smoking Cessation Counselling: Providing guidance and support to encourage and sustain smoking cessation. Development of a Written Action Plan: Prepared at discharge to guide patients in managing exacerbations effectively. Communication with the Patient’s Primary Care Physician: Writing a detailed letter to the family physician with updates on the patient’s condition and suggested management adjustments. Home-Based Patient Assessment: Evaluating the patient’s clinical status and environmental factors during home visits. Telephone Follow-Ups: Conducted at least once a month to monitor symptoms, provide guidance, and address any emerging concerns.	Nurses
[29] (2009) Ireland	The study aimed to evaluate the impact of a disease-specific home care (HC) programme on survival, exacerbation rates, and hospital admissions in patients with chronic obstructive pulmonary disease (COPD) requiring long-term oxygen therapy (LTOT), compared to standard care (SC).Cohort Study	Early discharge programme: - Number: 246. - Age: Mean age was 67 ± 9 years. Self-management education: - Number: 60. - Age: Mean age was 69 ± 9 years.	Disease Education: Provide comprehensive education about chronic obstructive pulmonary disease (COPD), focusing on its pathophysiology, progression, and impact on daily life. Smoking Cessation Counselling: Deliver targeted interventions to encourage and support smoking cessation, emphasising its critical role in disease management and stabilisation. Device Management: Educate patients on the correct use and maintenance of respiratory devices, including inhalers, nebulizers, and oxygen equipment, ensuring optimal therapeutic outcomes. Medication Monitoring and Collaboration: Collaborate with the medical team to monitor the patient’s condition and communicate any clinical changes that may require adjustments to treatment; support patients in adhering to prescribed medication regimens and educate them on the proper use, timing, and potential side effects of their medications. Chest Physiotherapy: Implement airway clearance techniques to improve ventilation, optimise lung function, and promote respiratory health. Individualised Home Exercise Programme: Develop and provide tailored exercise plans to enhance physical capacity and respiratory efficiency, designed according to the patient’s functional status and capabilities. Self-Management Plan: Collaborate with patients to create a personalised self-management plan, which includes recognising early symptoms of exacerbation; instructions on medication adjustments during exacerbations (e.g., self-administration of antibiotics, steroids, and bronchodilators); sputum clearance techniques; written guidance for early intervention; and strategies to prevent hospitalizations. Ongoing Monitoring and Rapid Access to Care: Ensure patients have scheduled follow-up appointments and maintain rapid access to respiratory outpatient clinics for timely evaluation and intervention during exacerbations. Telephone Support: Provide patients with access to telephone support for advice and reassurance, particularly during exacerbations, helping to address their concerns promptly and effectively.	Respiratory nurses; physiotherapists; and respiratory physicians
[30] (2009) Italy	The study aimed to evaluate the impact of a disease-specific home care (HC) programme on survival, exacerbation rates, and hospital admissions in patients with chronic obstructive pulmonary disease (COPD) requiring long-term oxygen therapy (LTOT), compared to standard care (SC). Cohort Study	- Number: 217 patients (108 HC group, 109 SC group). - Age: 68 ± 10 (HC); 66 ± 12 (SC). - Sex: 78% M (HC); 65% M (SC).	Disease Education: Educating patients and caregivers about chronic obstructive pulmonary disease (COPD), its progression, and management. Risk Factor Control: Reducing exposure to passive smoking, indoor and outdoor pollution, and infection risks. Body Weight Control: Supporting patients in maintaining an appropriate body weight to optimise respiratory function and overall health. Device Management: Training in the correct use and maintenance of medical devices, including oxygen therapy equipment. Medication Management: Supporting proper administration of medications, adherence to prescribed therapies, and adjustment when needed. Oxygen Administration Management: Ensuring correct use and compliance with long-term oxygen therapy (LTOT). Bronchial Drainage Techniques: Employing methods such as autogenic drainage, Positive Expiratory Pressure (PEP) mask, and expiration with the glottis open in the lateral posture (ELTGOL). Inspiratory Muscle Training: Implementing exercises to strengthen respiratory muscles, improving ventilation efficiency. Physical Training: Designing and guiding exercise programmes tailored to the patient’s needs, enhancing physical capacity and quality of life.	Respirator nurses, pneumologists, and rehabilitation therapists
[31] (2010 Australia	The study aimed to generate preliminary data to inform the design of a larger trial investigating the effects of the pulmonary maintenance programme on self-efficacy; functional exercise capacity; respiratory functioning; and quality of life in COPD patients. Quasi-Experimental Study	- Number: 29. - Age: Mean age was 71.7 ± 8.45 years. - Gender: 62.1% were men.	Collaborative Goal Setting: Nurses played a pivotal role in establishing individualised goals with the patient during the initial home visit. These goals were reviewed and adjusted at six and twelve months, ensuring alignment with the patient’s progress and evolving needs. Scheduled Telephone Follow-Ups: Nurses conducted regular telephone calls to monitor patient progress, provide tailored guidance, and reinforce adherence to the programme. These calls were scheduled weekly during the first month, and subsequently at two, five, eight, and ten months. Patient Education: Nurses provided continuous education on self-management strategies, disease progression, and techniques for preventing exacerbations. This education empowered patients to manage their condition more effectively and maintain the benefits of respiratory rehabilitation. Assessment of Symptom Perception and Functional Limitations: Nurses participated in monitoring functional and respiratory capacity using tools such as the Medical Research Council (MRC) Dyspnoea Scale and the Patient-Specific Functional Scale (PSFS). These assessments, conducted in collaboration with physiotherapists, tracked progress and identified any clinical changes. Psychosocial Support: Nurses addressed the emotional and psychological needs of patients, providing psychosocial support to alleviate anxiety and depression associated with COPD. This approach contributed to improved adherence and overall well-being. Promotion of Self-Efficacy: Nurses played a crucial role in promoting patients’ confidence in managing their respiratory challenges, adhering to the maintenance program, and performing daily activities. This intervention was embedded in telephone follow-ups and face-to-face interactions. Supervised Exercise Programme: Nurses were actively involved in supervising and encouraging adherence to the structured exercise program, which included warm-up exercises, preparing patients for more intense physical activity and reducing the risk of injury; strength training, focusing on muscle strengthening to enhance functional capacity; balance exercises aimed at improving stability and reducing the risk of falls; and stretching, promoting flexibility and preventing muscular stiffness. Nurses ensured correct and safe execution of these exercises during telephone follow-ups and in-person visits, supporting progression throughout the 12-month program.	Nurses and physiotherapists
[32] (2011) Turkey	To evaluate the impact of a nurse-led, home-based pulmonary rehabilitation programme on pulmonary function, arterial blood gases, quality of life, dyspnoea, and functional capacity in patients with advanced COPD. Quasi-Experimental Study	- Number: 32 patients divided equally between an intervention group (n = 16) and a control group (n = 16). - Age: The mean age was 71.8 ± 7.8 years in the intervention group and 65.1 ± 10.2 years in the control group.	Patient Education: Educational sessions were conducted 2–3 times in patients’ homes, lasting 2–3 h per session; information was provided both verbally and in pamphlets tailored to the patients’ specific symptoms. The content included the following: anatomy and physiology of the lungs; airway changes caused by COPD; causes and symptoms of the disease; instructions for the correct use of inhaled medications; breathing exercises, including pursed-lip and diaphragmatic breathing techniques; relaxation and breathing control methods; airway clearance techniques; modifications to daily living activities and energy conservation strategies; exercise recommendations; and smoking cessation methods. Nurses provided individualised education based on the patient’s needs, monitored comprehension, and distributed printed materials. Exercise Programme: The exercise programme included three components: Lower Extremity Aerobic Exercise (patients were instructed to walk for 30 min daily, adjusting the pace to their individual abilities) and Upper Extremity Aerobic Exercise (a variety of arm exercises were performed, each for 15 repetitions per set, with each set lasting at least 1 min). Examples of exercises included the following: stirring the soup in a cauldron; pulling up the anchor; hitting the punching bag; rowing the boat; marching arm movements; punching; waxing the car (“wax on, wax off”); performing the crawl and breaststroke movements; playing the drums and accordion; chopping wood; picking cherries; pulling weeds; wiping the windshield; stretching the springs; and scissoring the arms. Patients could complete exercises in intervals if needed. Breathing Exercises: Pursed-lip breathing and diaphragmatic breathing were practiced for 30 min each day. Nurses demonstrated these exercises during the first session and provided ongoing supervision. Home-Based Implementation and Monitoring: Patients were instructed to perform the prescribed exercises independently at home and to maintain a record of any challenges encountered. Nurses monitored progress and adherence through follow-up visits, where exercise performance was evaluated; addressing challenges identified during the visits and correcting improper exercise techniques; and phone calls made every 15 days during the first month and monthly in subsequent months to ensure adherence. Supervision and Evaluation: During the second and third home visits, nurses observed patients performing the exercises, evaluated their execution, and addressed any issues identified. Nurses played a key role in reinforcing the correct techniques for breathing and physical exercises to optimise outcomes.	Nurses
[33] (2016) Turkey	The primary aim was to determine whether home nursing care based on Orem’s self-care model enhances the self-efficacy of patients with COPD. The secondary goal was to assess the impact of this intervention on symptom management, including dyspnoea and related complications. Quasi-Experimental Study	The study involved 106 patients diagnosed with COPD in northern Turkey. The participants were divided into two groups: intervention (n = 53) and control (n = 53). - Age (Mean ± SD): 65.1 ± 8.4 years. - Gender Distribution: 62.3% male and 37.7% female.	Education about COPD: Educate patients and their families about the definition, causes, symptoms, and diagnostic methods of chronic obstructive pulmonary disease (COPD); this information was provided during home visits and through the distribution of an educational guide developed specifically for patients and their families. Guidance on COPD Management: Provide strategies for managing COPD symptoms, including techniques to alleviate dyspnoea, reduce fatigue, and improve activity tolerance; these strategies were integrated into the individualised care plans developed during the home visits. Nutritional Counselling: Offer guidance on proper nutrition to prevent imbalanced nutrition and ensure patients meet their dietary needs, as outlined in the care plan. Respiratory Exercises: Teach and supervise patients in performing diaphragmatic breathing and pursed-lip breathing to improve respiratory efficiency and control dyspnoea; these exercises were explicitly mentioned in the educational guide and reinforced during home visits. Smoking Cessation Support: Educate and motivate patients to quit smoking, emphasising its role in slowing disease progression and improving quality of life; smoking cessation was a key focus in the educational materials provided to patients. Medication Adherence: Educate patients on the importance of regular and effective use of prescribed medications for symptom control and disease management; this topic was addressed during the educational sessions as part of the care plan. Prevention of Respiratory Infections: Provide education on protecting the upper respiratory system from infections, including hygiene practices and effective coughing techniques; these strategies were included in the educational guide distributed to patients and families. Emotional and Psychological Support: Offer support to patients experiencing anxiety or psychological distress due to COPD, improving their emotional state and motivation for self-care; emotional support was part of the holistic approach to care provided during home visits.	Nurses
[34] (2020) United States	The primary purpose of the study was to assess whether the implementation of a COPD self-management care plan packet in home healthcare could reduce the rate of 30-day rehospitalizations among patients with COPD. Secondary objectives included improving COPD symptom management and medication reconciliation rates, as well as promoting timely follow-up with healthcare providers. Quasi-Experimental Study	- Number: Preintervention sample: 33 patients; Postintervention sample: 26 patients. - Age: Preintervention: 51–96 years (Mean ± SD: 75 ± not reported); Postintervention: 55–92 years (Mean ± SD: 77 ± not reported). - Gender Distribution: Preintervention: 14 males, 19 females; Postintervention: 12 males, 14 females.	Identification of Patient-Specific Learning Needs: Nurses used a daily form to assess and document patient-specific learning needs, such as adherence to prescribed medication therapy, including oxygen therapy; dietary and lifestyle modifications to manage symptoms; and smoking cessation. This form was designed to support daily self-management and was reviewed during each nursing visit to adjust care plans based on the patient’s progress. Educational Interventions Through Informative Handouts: Educational sessions were conducted by nurses using targeted handouts that focused on key aspects of COPD self-management: the purpose and proper technique for using inhalers; safety guidelines for oxygen therapy; and smoking cessation education to encourage and support patients in quitting smoking. Lifestyle and Symptom Management Education: Nurses provided additional educational materials and guidance to patients, addressing lifestyle modifications and symptom management strategies; identifying and avoiding symptom triggers; performing deep breathing exercises to improve respiratory function; engaging in daily activities as tolerated by the patient’s physical condition; maintaining a healthy and balanced diet to support overall well-being; and recognising and managing anxiety and/or depression, which are often associated with COPD. These materials were stored in the patient’s home for daily use and were reviewed during nursing visits to ensure proper application. Development of a COPD-Specific Action Plan: Nurses collaborated with patients to create a COPD action plan, which included the following: a daily symptom log for tracking symptoms and oxygen saturation levels; instructions categorised into “zones” (green for controlled symptoms, yellow for worsening symptoms, and red for emergent symptoms) to guide patient decision-making. The action plan empowered patients to manage their symptoms effectively and provided clear instructions for seeking help when symptoms worsened. Medication Reconciliation: During admission, nurses conducted a thorough medication reconciliation to ensure accuracy in prescribed COPD medications and addressed any deficiencies. Coordination of Timely Follow-Up Care: Nurses ensured that patients had follow-up appointments scheduled with their primary care providers or pulmonologists within one week of hospital discharge, promoting continuity of care and reducing readmission risks. Reinforcement of COPD Self-Management During Visits: During each nursing visit, nurses reviewed the patient’s self-management progress; the materials and action plans provided during previous sessions; and proper inhaler techniques, lifestyle modifications, and symptom management strategies. Assessment of COPD Symptoms Using the COPD Assessment Test (CAT): Nurses utilised the CAT tool to assess the severity of COPD symptoms during admission and discharge. The results were used to evaluate the patient’s risk of readmission and the effectiveness of the interventions.	Nurses

**Table 5:** Quality assessment for cohort studies.

	Lawlor et al., (2009) [29]	Rizzi et al., (2009) [30]
Were the two groups similar and recruited from the same population?	YES	YES
Were the exposures measured similarly to assign people to both exposed and unexposed groups?	YES	YES
Was the exposure measured in a valid and reliable way?	YES	YES
Were confounding factors identified?	NO	YES
Were strategies to deal with confounding factors stated?	NO	YES
Were the groups/participants free of the outcome at the start of the study (or at the moment of exposure)?	YES	YES
Were the outcomes measured in a valid and reliable way?	YES	YES
Was the follow-up time reported and sufficient to be long enough for outcomes to occur?	YES	YES
Was follow-up complete, and if not, were the reasons for loss to follow-up described and explored?	YES	YES
Were strategies used to address incomplete follow-up?	NO	YES
Was appropriate statistical analysis used?	YES	YES

**Table 6:** Quality assessment for RCT studies.

	Hermiz et al. (2002) [27]	Coultas et al. (2005) [28]
Was true randomization used for the assignment of participants to treatment groups?	YES	YES
Was allocation to treatment groups concealed?	NO	NO
Were treatment groups similar at the baseline?	YES	YES
Were participants blind to treatment assignment?	NO	NO
Were those delivering the treatment blind to treatment assignment?	NO	NO
Were treatment groups treated identically other than the intervention of interest?	NO	YES
Were outcome assessors blind to treatment assignment?	Unclear	YES
Were outcomes measured in the same way for treatment groups?	YES	YES
Were outcomes measured in a reliable way?	YES	YES
Was follow up complete, and if not, were differences between groups in terms of their follow up adequately described and analysed?	YES	YES
Were participants analysed in the groups to which they were randomised?	YES	YES
Was appropriate statistical analysis used?	YES	YES
Was the trial design appropriate and any deviations from the standard RCT design (individual randomization, parallel groups) accounted for in the conduct and analysis of the trial?	No	Yes

**Table 7:** Quality assessment for quasi-experimental studies.

	Cooke et al., (2009) [31]	Akinci et al., (2011) [32]	Bal Özkaptan et al., (2016) [33]	McGill et al., (2020) [34]
Is it clear in the study what is the “cause” and what is the “effect” (i.e., there is no confusion about which variable comes first)?	YES	YES	YES	YES
Was there a control group?	NO	YES	YES	NO
Were participants included in any similar comparisons?	YES	YES	YES	YES
Were the participants included in any comparisons receiving similar treatment/care other than the exposure or intervention of interest?	YES	YES	YES	YES
Were there multiple measurements of the outcome, both pre- and post-intervention/exposure?	YES	YES	YES	YES
Were the outcomes of participants included in any comparisons measured in the same way?	YES	YES	YES	YES
Were outcomes measured in a reliable way?	YES	YES	YES	YES
Was follow-up complete and if not, were differences between groups in terms of their follow-up adequately described and analysed?	NO	NO	YES	NO
Was appropriate statistical analysis used?	YES	YES	YES	YES

**Table 8:** Types of interventions in pulmonary rehabilitation for older adults with COPD in a home setting.

		[27]	[28]	[29]	[30]	[31]	[32]	[33]	[34]
Health Education	About the condition	✓	✓	M	M	✓	✓	✓	✓
	Correct use of medication (especially inhalers)	✓	✓	M	M		✓	✓	✓
	Smoking cessation	✓	✓	M	M		✓	✓	✓
	Symptom and exacerbation management	✓	✓	M		✓	✓	✓	✓
	Nutritional counselling	✓			M			✓	✓
Respiratory Training and Physical Exercise	Only education on physical exercise	✓							
	Aerobic and/or strength training			M	M		✓		
	Respiratory exercises			M	M		✓	✓	
	Airway clearance techniques			M	M		✓	✓	
	Education about energy conservation techniques	✓					✓	✓	✓
Tailor-Made Plan, Monitoring, and Follow-up		✓	✓	M	M	✓	✓	✓	✓
Psychological Support		✓	✓			✓	✓	✓	✓
Oxygen Therapy Management				M	M				✓

## Data Availability

Data are available only upon request to the authors.
